# A Comparison Study Between Electrical Muscle Stimulation and Transcutaneous Electrical Nerve Stimulation on Treatment of Myofascial Pain Syndrome

**DOI:** 10.1111/aor.70017

**Published:** 2025-10-04

**Authors:** Seekaow Churproong, Benjamin Metcalfe, Polly Mcguigan, Dingguo Zhang

**Affiliations:** ^1^ Department of Electronic and Electrical Engineering University of Bath Bath UK; ^2^ Institute of Medicine Suranaree University of Technology Nakhon Ratchasima Thailand; ^3^ Department of Health University of Bath Bath UK

**Keywords:** electrical muscle stimulation, muscle stretch, myofascial pain syndrome, sham stimulation, transcutaneous electrical nerve stimulation

## Abstract

**Background:**

Myofascial pain syndrome (MPS) originates from myofascial trigger points (MTPs)– hypersensitive nodules commonly found in the trapezius muscle (TM) that cause pain and functional limitations. While transcutaneous electrical nerve stimulation (TENS) is a conventional treatment, a novel approach combining electrical muscle stimulation (EMS) with active stretching (AS) has recently been developed (EMS + AS).

**Methods:**

EMS electrodes were placed transversely across muscle fibers to induce localized contractions and thus greater stretch of MTP‐containing regions compared to AS alone. EMS plays a role similar to a therapist's hand in passive stretching in that it provides resistance force. Forty‐one participants with MTPs in the TM received single sessions of EMS + AS, sham stimulation (SS) + AS, and TENS. Each session included three 10‐s stimulations with 10‐s rest intervals. Pain intensity (PI), pressure pain threshold (PPT), and surface electromyography (sEMG) for maximal voluntary contraction (%MVC) amplitude analysis of TM function improvement were the three outcome measures used to assess treatment effectiveness. To evaluate the immediate effects of short‐duration treatments with EMS + AS compared to SS + AS and TENS. All three treatments were applied in a randomized order.

**Results:**

EMS + AS showed significant improvements in PI and PPT (*t*_(40)_ = −6.01 and *t*_(40)_ = 5.38, *p* < 0.001, respectively). EMS + AS showed a small sEMG activity during TM function improvement of 0.49 ± 0.056 %MVC at post‐treatment, normalized to pre‐treatment values. Compared to SS + AS and TENS, EMS + AS significantly increased PPT changes (*F*_(2,120)_ = 13.442, *p* < 0.001); however, there were no significant differences in PI or mean %MVC.

**Conclusions:**

This study demonstrates that EMS generates a local contraction instead of a full contraction for a muscle. EMS's effect is related to the aim of mimicking passive stretching performed by the therapist's hand. Ultimately, EMS + AS has the potential to be an effective approach for alleviating MPS symptoms.

## Graphical abstract

Transverse placement of electrical muscle stimulation (EMS) electrodes across muscle fibers elicits localized contractions, facilitating greater active stretching (AS) of regions containing myofascial trigger points. This EMS + AS approach mimics passive stretching, with EMS providing resistance force similar to that typically applied by a therapist's hand.

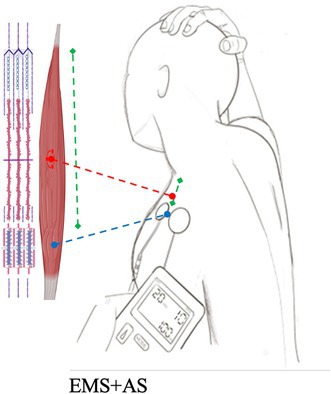

**Abbreviations**
AC: alternating current
AS: active stretching
BMI: body mass index
DC: direct current
EMS + AS: a combination treatment of electrical muscle stimulation and active stretching
EMS: electrical muscle stimulation
GTOs: Golgi tendon organs
Hz: hertz
IED: inter‐electrode distance
kgf: kilogram‐force
mA: milliampere
MD: mean difference
MPS: myofascial pain syndrome
MTPs: myofascial trigger points
mV: millivoltage
MVC: maximal voluntary contraction
NRS: numeric rating scale
PI: pain intensity
PPT: pressure pain threshold
PS: passive stretching
ROM: range of motion
SD: standard deviation
sEMG: surface electromyography
SNR: signal‐to‐noise ratio
SS + AS: a combination treatment of sham stimulation and active stretching
SS: sham stimulation
TENS: transcutaneous electrical nerve stimulation
TM: trapezius muscle
μs: microseconds

## 1 Introduction

Pain is a complex experience with both emotional and physical dimensions. Chronic pain and the severity of pain intensity (PI) can significantly impact the quality of life [1]. The pain component of myofascial pain syndrome (MPS) significantly impacts work function [2]. Despite limited access to diagnostic facilities, the reported prevalence of MPS has substantial health and socioeconomic consequences [3]. In local muscle tissue, fluid accumulation from micro‐injuries can lead to the release of waste products and inflammatory debris, potentially resulting in fibrosis [4]. The irritable fibrotic nodules within taut muscle bands are called myofascial trigger points (MTPs). Pain recognition at MTP sites, local tenderness, a local twitch response, and palpation of the snapping nodule are common diagnostic methods for MPS. Latent MTPs cause pain only under pressure, and active MTPs cause spontaneous pain [5, 6, 7]. MTP sensitivity is typically assessed using the pressure pain threshold (PPT) [8]. MTPs within the trapezius muscle (TM) can significantly contribute to muscular neck and shoulder pain, affecting TM function by limiting shoulder elevation in the upper TM and scapular retraction in the middle TM. MTPs are prevalent in both upper and middle TM segments [9]. This study aimed to investigate PI, PPT, and muscle electrical activity during TM function in adults with MTPs in both TM regions. Surface electromyography (sEMG) signals during TM function can be evaluated using amplitude analysis, quantified as a percentage of maximal voluntary contraction (%MVC) [10].

Several treatments have demonstrated long‐term benefits in managing MPS through pain‐reducing mechanisms [5, 11, 12] but often require significant time commitments. For instance, Transcutaneous Electrical Nerve Stimulation (TENS), a popular portable modality, alleviates pain by stimulating non‐nociceptive fibers through the gate control theory and releasing analgesic neurochemicals [13, 14]. However, TENS typically requires 5 to 30 min of treatment for immediate pain relief [14]. Due to the long duration of treatment, working adults prefer not to seek treatment despite their suffering [15]. Our study addresses this challenge by presenting a novel, non‐invasive, one‐minute electrical stimulation treatment. We combined Electrical Muscle Stimulation (EMS) with Active Stretching (AS) of the TM, offering an alternative MPS treatment that delivers short‐term benefits. This suggests that with continued application, long‐term effectiveness may be achievable. The EMS + AS approach for MPS relief intends to mimic therapist‐applied passive stretching (PS). EMS aims to produce an effect similar to the therapist's hand during PS.

### 1.1 Review of TENS and EMS Applications in MPS

TENS is safe to use even in older populations with no serious side effects, appearing to reduce pain more effectively than placebo [16, 17]. TENS provides immediate pain relief in MTPs of the neck and upper back [18]. The effects of TENS on MPS significantly decrease PI, but did not significantly increase PPT with conventional TENS [19]. Conventional TENS or TENS with a frequency greater than 50 Hz, typically used at lower intensity levels, has a more rapid onset of pain relief than TENS with a frequency less than 20 Hz [20], even though there was no statistically significant difference in pain outcomes [16]. A high‐frequency‐low‐intensity TENS mode aimed to stimulate peripheral nerves for pain relief. This mode of stimulation typically results in tingling sensations without twitching. Electrical signals can interrupt pain transmission to the brain [13].

Unlike TENS, EMS primarily targets muscle contraction but is not commonly applied to alleviate myofascial pain. However, previous studies demonstrated the acute effects of EMS on MPS [21, 22, 23]. While neuromuscular electrical stimulation (NMES) aims to induce muscle contraction for rehabilitation, EMS refers more broadly to the use of stimulation to activate muscles, and our use of EMS aligns with previous research and terminology of Hsueh et al. that compared EMS effects with TENS and sham stimulation (SS) using established research protocols [22, 23]. For both TENS and EMS modalities, the cathode is placed on the MTP, with the anode positioned near the acromion. TENS uses a frequency of stimulation at 60 Hz, while EMS uses a frequency of stimulation at 10 Hz for 20‐min MPS treatments. Both EMS and TENS improved PI, PPT, and range of motion (ROM) when compared to SS. However, this EMS method showed only a significant ROM improvement but did not demonstrate a significant difference in PI and PPT when compared to TENS [14, 22]. While a 20‐min treatment of TENS and EMS offered immediate relief for upper TM trigger points, the next research established a similar treatment protocol for long‐lasting benefits, which were additionally evaluated after 2 weeks and 3 months. Both groups of EMS and TENS, which received continuous treatment, showed significant improvement in pain outcomes. Participants in the control group were instructed to stretch the upper TM for ten seconds, three times per day, as described in previous studies [23]. However, this study indicates that self‐stretching alone or AS of the TM did not have enough potential to alter pain outcomes and MTP pathology.

### 1.2 Review of Stretching Therapy in MPS

Basic stretching exercises rely on two sources of resistance during the stretch: passive structural stiffness (from the muscle and connective tissue), which resists stretching, and tonic reflex activity (from peripheral and central origins). Static stretching involves holding a muscle in an extended position at mild tension for 15 to 60 s. Static stretches can be categorized into two types: active stretching (AS), performed independently, and passive stretching (PS), which requires external assistance. The Force‐Length relationship is crucial to understanding how stretching works, dictating that muscle tension diminishes when sarcomeres, the basic contractile units of muscle, are excessively lengthened or shortened [24]. Muscle stretching reduces tension by increasing sarcomere length and potentially disrupting cross‐bridge formations, leading to muscle relaxation and pain relief. For AS, individuals typically execute a few TM stretches for each set within 10–20 s [25]. PS also requires minimal time but has been shown to be more effective than AS for immediate gains in flexibility [26]. Despite its effectiveness, some individuals have reported discomfort during therapist‐assisted PS because full muscle relaxation may not always occur [27]. Therefore, this study aimed to compare the acute effects of a novel EMS + AS intervention to SS + AS and TENS. We hypothesized that EMS + AS would demonstrate superior improvements in PI, PPT, and %MVC amplitude analysis compared to the other two modalities. PI and PPT served as our primary outcome measures, assessing changes in pain perception and mechanical pain sensitivity. %MVC amplitude analysis was the secondary outcome, used to evaluate improvements in sEMG activity during upper or middle TM function.

## 2 Methods

**FIGURE 1:**
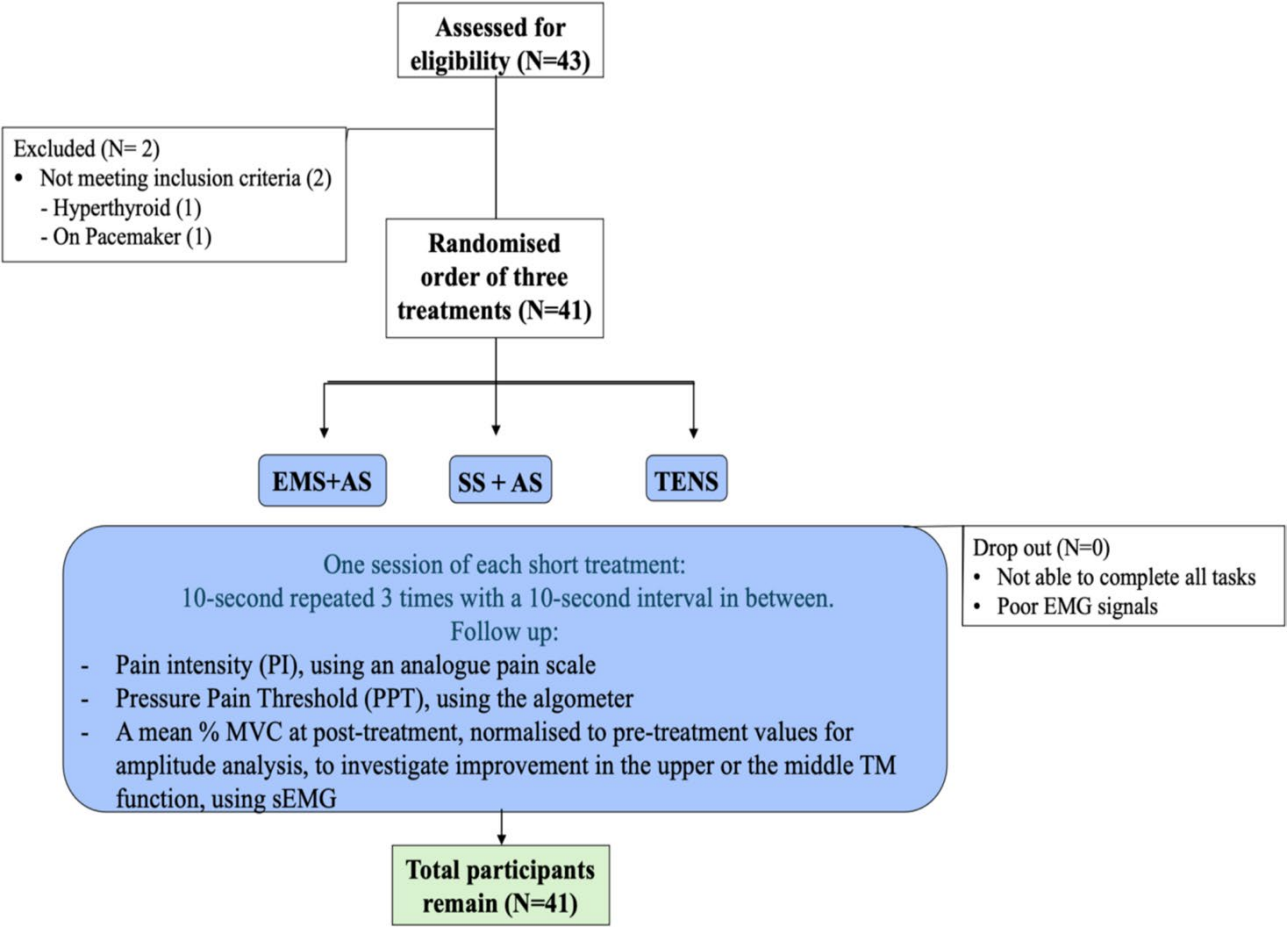
Flow of the study diagram. [Color figure can be viewed at wileyonlinelibrary.com]

The study received approval from the University of Bath's Research Ethics Approval Committee for Health (approval reference EP 22081). Participants were included if meeting the inclusion criteria. Inclusion criteria were: age 18 or older, self‐reported upper back pain, a physician's diagnosis of MPS with trigger points in the TM, absence of chronic diseases (e.g., advanced cancer, cardiovascular diseases, stroke, pulmonary embolism, heart arrhythmia, cervical nerve root compression, spinal cord injury, muscle weakness, seizure or epilepsy, skin ulcer or any skin infection on the upper back, hypo‐ and hyperthyroid, autoimmune diseases), ability to move upper extremities, no use of pacemakers or long‐acting analgesic drugs, and no history of allergies to materials such as metallics, gel pads, or alcohol cleansing agents. During the screening stage, 43 working adults applied for this study; two were excluded due to uncontrolled heart rhythms related to hyperthyroidism, and one for having a pacemaker. Thus, 41 participants meeting the inclusion criteria were enrolled and instructed to avoid skin irritants, activities causing muscle soreness, and narcotic use prior to the experiment. The study was conducted in an isolated room. An experienced physician (over 10 years in MTP diagnosis and treatment) re‐evaluated the presence of MTPs through physical examination. The number and location of MTPs in the upper or middle TM were recorded. Each participant received three treatments—EMS + AS, SS + AS, and TENS—administered in a single session and applied in randomized order. Changes in PI and PPT were monitored for each treatment. Three sEMG recordings of maximal voluntary TM contractions were used for amplitude analysis (%MVC) for each treatment. The study design flow diagram is shown in Figure 1.

### 2.1 Treatment Protocol

Participants were seated on a chair throughout the experiment. After marking the MTP on the TM, the local skin was cleaned with alcohol wipes, and hair was shaved from the targeted upper and middle TM sections. Proper skin preparation minimizes electrode‐skin impedance, ensuring optimal current delivery during the treatment sessions. Participants received three treatments in random order:

1. **EMS + AS**: Participants received EMS using a Premier Rechargeable Digital EMS Muscle Stimulator (EM‐6200A) from Med‐Fit. The device features dual isolated channels, each of which can deliver an asymmetrical bi‐phasic square pulse of intensity 0–100 mA peak (maximum voltage 50 V). We employed a frequency of 20 Hz and a pulse width of 100 μs. The current intensity was individually adjusted to achieve comfortable tetanic contractions, transversely placed electrodes with a small inter‐electrode distance (IED).
  - **Upper TM**: Two round electrodes (diameter 32 mm, 1 cm IED) were placed near the acromion and adjacent to the MTP.
  - **Middle TM**: Two rectangular electrodes (20 × 40 mm, 1 cm IED) were placed near the spine, opposite the stretch direction.

EMS was applied for 3 s without AS, followed by 10 s of AS while EMS was operating (the subject volitionally depressed the shoulder for upper TM or protracted the scapula for middle TM). The protocol repeated three AS for treatment with three 10‐s rest intervals to a total of 60 s (this duration did not include the 3 s of EMS application without AS). This EMS + AS approach aimed to target localized muscle contraction and enhance TM stretching while avoiding direct trigger point stimulation.

2 **SS + AS**: Participants performed AS using the same EMS device and electrode placement as in the EMS + AS protocol. However, no electrical current was applied. The procedure started with a conversation about the treatment from the researcher while the current amplitude was reduced from 1 mA to 0 mA during TM stretching, accompanied by a beeping sound to simulate stimulation. Participants were informed that this was the intended treatment. SS + AS represented AS alone.
3 **TENS**: Conventional TENS (60 Hz, 100 μs, 5 mA) was applied using two electrodes placed directly over the MTP region. The TENS protocol was adapted from previous studies investigating TENS and EMS for MPS [21, 22, 23]. The TENS approach was used without TM stretching, and we did not observe muscle contraction, assuming TENS demonstrated only a weak contraction effect [13].

**FIGURE 2:**
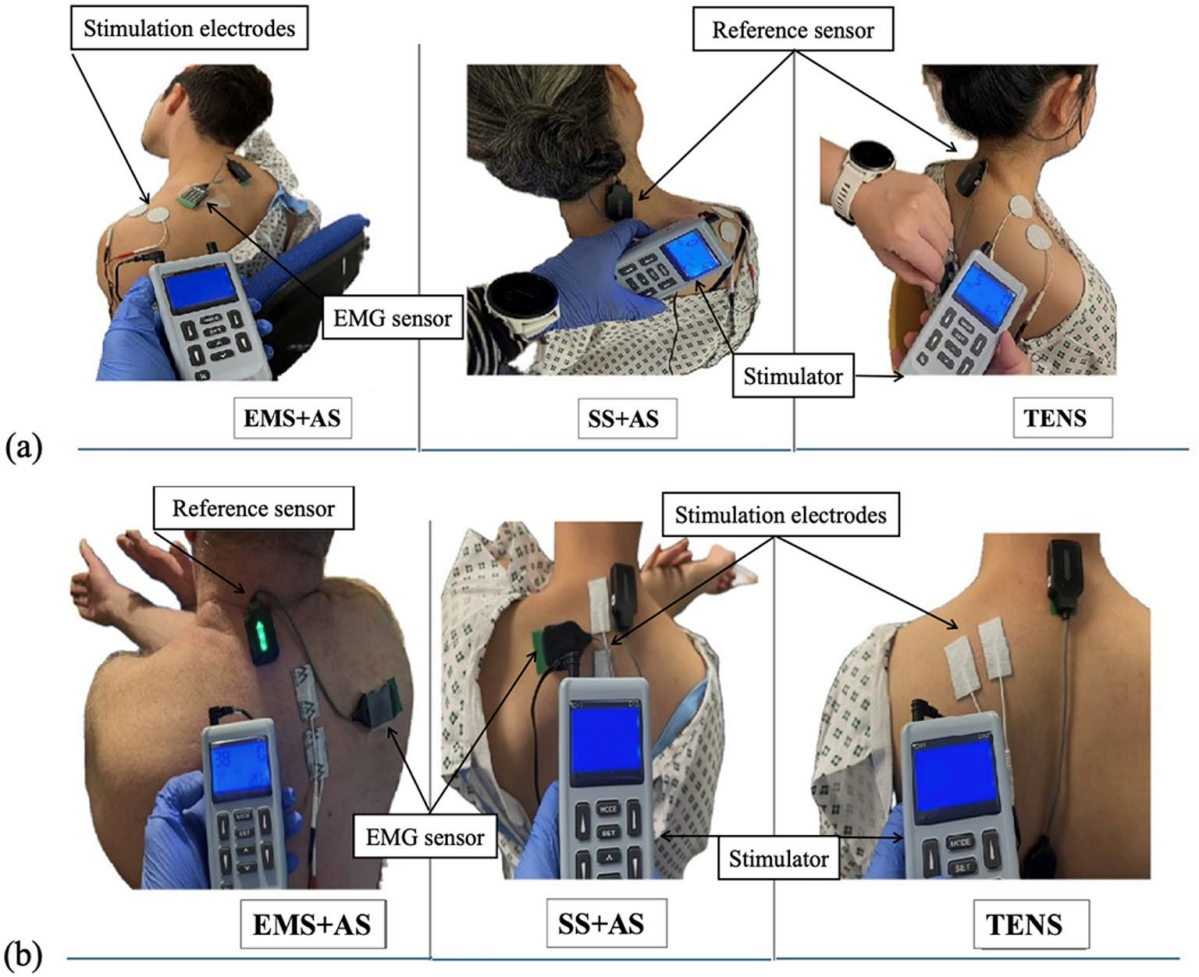
Examples of the three treatments applied to (a) the upper TM and (b) the middle TM. [Color figure can be viewed at wileyonlinelibrary.com]

The same protocol was used for all three treatments, consisting of three 10‐s stretching periods with 10‐s rest intervals, followed by a 2‐min break. The treatment order was randomized using computer‐generated serial numbers. Participants received guidance from the therapist. The procedures for the three treatments on the upper and middle TM in this study are shown in Figure 2.

### 2.2 Pain Assessment

This study assessed pain outcomes as follows:

1. Participants self‐reported their PI at the MTPs using a numeric rating scale (NRS) ranging from 0 (no pain) to 10 (worst pain) [14, 19].
2. A digital pressure algometer (Wagner instrument, Greenwich, USA) was used to measure the peak compression force applied to the MTP using a 1 cm^2^ round‐shaped rubber disk for PPT (demonstrating high intra‐rater reliability). The MTP was identified through gentle and firm palpation, and the rubber‐tipped load handle was then placed over the MTP. A steady force was applied until the participant reported the first sensation of pain, at which point the value was recorded and reported in kilogram‐force (kgf) [8].

### 2.3 Muscle Electrical Activity Assessment

The sEMG system (Trigno Maize Sensor, Delsys company) incorporated 16 channels; 2 mm diameter silver dry sensors (27 × 37 × 13 mm, 20 g, 6 mm IED) were used. Four channels (5 × 1 mm) were specifically used for the reference electrode. Sensors captured 17‐bit resolution data at 1000 samples/s (20–450 Hz bandwidth), optimized for 83–1000 mV DC offset and 15.6–20.8 mV AC sEMG signals using Trigno Discover software (v1.6.5) [28]. Filtering reduced noise, suitable for superficial muscles such as the TM [29]. Given the inconsistent activation patterns of the TM during intense contractions [30], and the potential benefits of sEMG in trigger point research, sensors were placed directly on MTPs, typically located on the muscle belly, for precise data acquisition. The EMG application steps were as follows:

1. The MTP was marked on the TM while participants sat with arms resting at their sides.
2. Skin was gently scrubbed with non‐abrasive, sandpaper‐like crystal pads to remove hair and debris, then cleaned with alcohol.
3. The Trigno Maize Sensor was initially placed on the MTP for pre‐test recording. The EMG sensor was then removed during TENS or EMS to allow electrical stimulation of the MTP. The EMG sensor was carefully repositioned to the marked locations after each treatment for post‐test recording. The reference sensor was positioned on the C7 spinous process [29] due to minimal movement (Figure 2). The sensor was securely attached using adhesive.
4. Data Normalization:
  - MVC was established using a standardized protocol (shoulder shrug for upper TM, scapular retraction for middle TM).
  - Data were normalized to pre‐maximal EMG data during full shoulder shrug or scapular retraction for each participant, allowing for the reporting of changes in %MVC after treatments [31].
5. Data Acquisition:
  - Three sets of maximum TM actions were performed with 1–2 s per action.
  - Participants practised shoulder elevation or scapular retraction before recording.
  - Data were collected immediately before and after each treatment.

**FIGURE 3:**
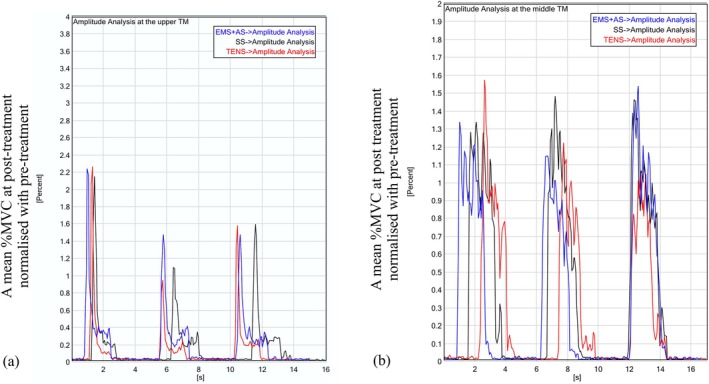
Examples of EMG amplitude analysis of three trapezius muscle (TM) actions for three treatments: EMS + AS (blue line), SS + AS (black line), and TENS (red line), applied to (a) the upper TM and (b) the middle TM. [Color figure can be viewed at wileyonlinelibrary.com]

The percentage change of EMG was relative to the pre‐treatment (baseline), using changes in %MVC after treatment. For more accurate data, EMG signals from 16 channels, which were selected based on their signal‐to‐noise ratio (SNR), were averaged, and raw EMG data were imported into EMG Works analysis software version 4. A smart threshold function identified the time windows of three TM actions for post‐treatment amplitude analysis. Post‐treatment values were normalized to pre‐treatment values on a channel‐by‐channel basis to calculate %MVC [8, 28]. During the 1–2 s period of TM action, as specified in the protocol, EMG data were quantified using the time‐amplitude integral of the root mean square (RMS), expressed as a percentage of the rectified mean %MVC. The average %MVC across the three actions was calculated for comparison between each treatment. Amplitude analysis examples are shown in Figure 3.

This study assessed PI and PPT before and after each treatment. Mean and standard deviations (SD) were calculated for all participants, with statistical significance set at *p* < 0.05. Paired *t*‐tests compared pre‐ and post‐treatment PI and PPT values. One‐way ANOVA compared post‐treatment changes in PI, PPT, and mean %MVC across the three treatments, normalized to pre‐treatment values. Given the normal distribution of the data, a Bonferroni post hoc test was conducted subsequent to ANOVA for multiple‐group comparisons.

## 3 Results

This study included 41 participants (15 males, 26 females) with a mean age of 38.83 ± 12.81 years and BMI of 24.47 ± 5.24 kg/m^2^. Participant demographics and clinical characteristics, including symptom duration, overall pain intensity, and MTP locations, are presented in Table 1. The study identified 30 latent MTPs and 11 active MTPs. All right‐hand dominant participants were from diverse occupations, including university students.

**TABLE 1:** Descriptive statistics characteristic.

Item	Total (*N* = 41)
Age (years); Mean (SD)	38.83 ± 12.81
BMI (kg/m^2^); Mean (SD)	24.47 ± 5.24
Gender	
Male (%)	16 (36.6%)
Female (%)	25 (63.4%)
Ethnicity	
Asian background (%)	29 (70.7%)
White (European, British) (%)	11 (26.8%)
Other (e.g., Arabs, etc.) (%)	1 (2.4%)
Occupations	
University students (%)	17 (41.5%)
Business work (manager, owner, etc.) (%)	8 (19.5%)
Computer work (programmer, etc.) (%)	5 (12.2%)
Kitchen work (chef, baker) (%)	5 (12.2%)
Hospital work (PT, nurse, etc.) (%)	3 (7.3%)
Nursery work (nanny, cleaner) (%)	3 (7.3%)
Type of job task	
Repetitive (%)	25 (61.0%)
Mixed (%)	13 (31.7%)
Awkward (%)	3 (7.3%)
Type of MTPs	
Active (%)	11 (26.8%)
Latent (%)	30 (73.2%)
GP informed (years); Mean (SD)	2.11 ± 2.30
Duration of symptoms (years); Mean (SD)	2.34 ± 3.66
Using technology devices (hours/day); Mean (SD)	6.71 ± 3.14
Pain intensity (overall); Mean (SD)	6.22 ± 1.33
Pain location	
Right side of TM	20 (48.8%)
Right and Left sides of TM	17 (41.5%)
Left side of TM	4 (9.8%)

The average EMS amplitude for upper and middle TM sections was 23.46 ± 12.82 mA, while the specific amplitude of the TENS group was 5 mA. Paired *t*‐tests revealed significant reductions in PI after all treatments (Table 2). EMS + AS demonstrated significant improvement in both PI (*t*_(40)_ = −6.01, *p* < 0.001) and PPT (*t*_(40)_ = 5.38, *p* < 0.001). Neither SS + AS nor TENS showed significant changes in PPT.

**TABLE 2:** Changes in PI and PPT between before and after treatments.

Task	Change in PI MEAN ± SD	*t*	Change in PPT MEAN ± SD (kg/f)	*t*
EMS + AS	−1.05 ± 1.12	−6.01**	0.38 ± 0.46	5.38**
2 SS + AS	−0.54 ± 1.29	−2.67*	−0.07 ± 0.41	−1.09
3 TENS	−0.68 ± 0.88	−4.98**	0.01 ± 0.40	0.19

* *p* < 0.05.

** *p* < 0.001.

**FIGURE 4:**
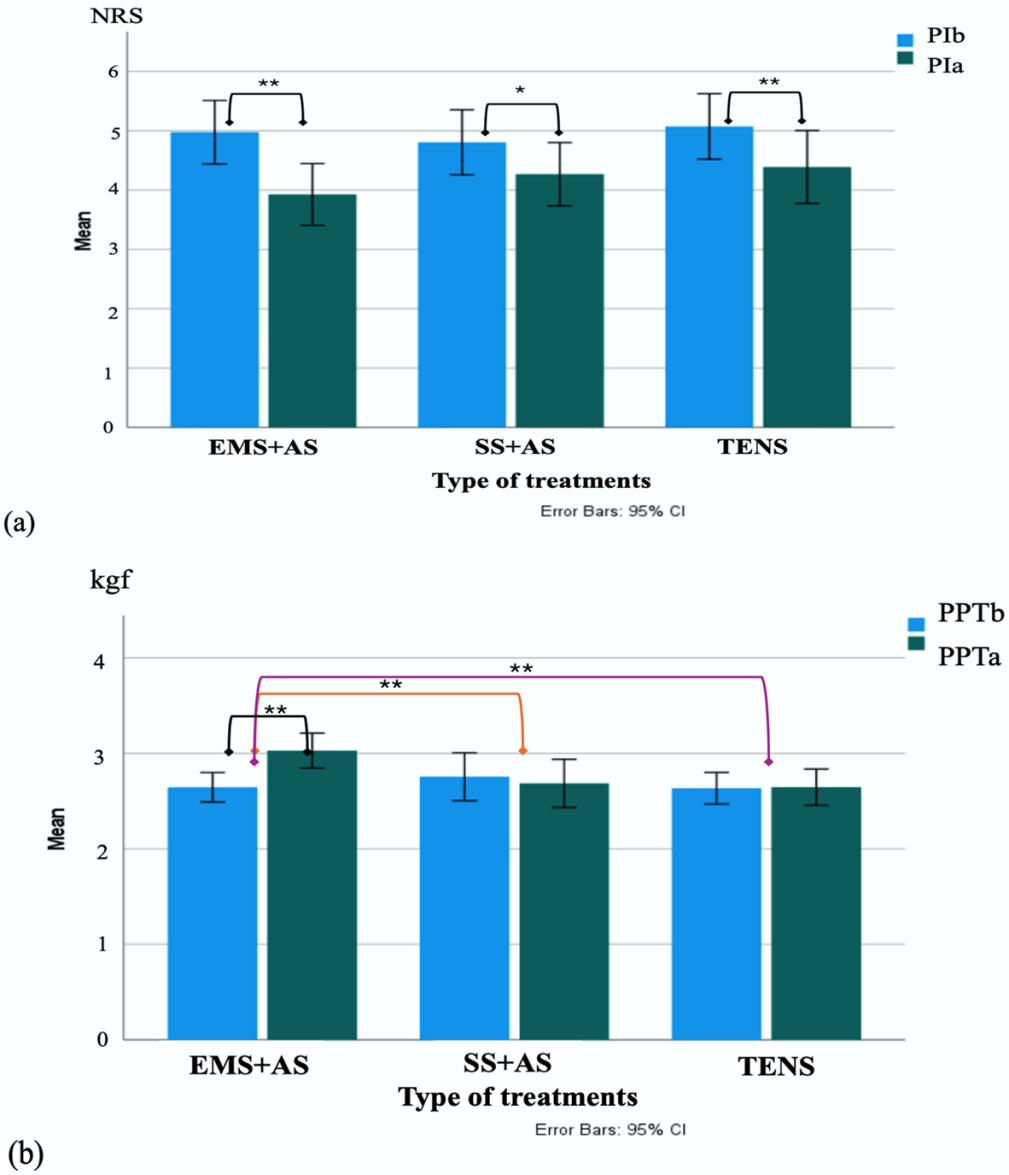
(a) Illustration comparing the mean pain intensity (PI) before (PIb) and after (PIa) treatment for each group, measured using numeric rating scale (NRS). The black line indicates a significant decrease in PI after EMS + AS, SS + AS, and TENS treatments. (b) Illustration comparing the mean pressure pain threshold (PPT) before (PPTb) and after (PPTa) treatment for each group, measured in kilogram‐force (kgf). The black line indicates a significant increase in PPT after EMS + AS treatment. Orange and purple lines indicate significantly greater PPT changes with EMS + AS compared to SS + AS and TENS, respectively (*p < 0.05, **p < 0.001). [Color figure can be viewed at wileyonlinelibrary.com]

One‐way ANOVA revealed a significant difference in PPT among the three treatments, *F*_(2,120)_ = 13.442, *p* = < 0.001, *η* ^2^ = 0.183 (95% CI 0.067–0.294), indicating meaningful differences among treatments. Bonferroni post hoc test showed EMS + AS had significantly higher PPT than SS + AS (MD = 0.454 kgf, *p* < 0.001) and TENS (MD = 0.373 kgf, *p* < 0.001). No significant differences were found among treatments for PI between TENS and SS + AS. Multiple comparisons of the mean PI and PPT for each treatment before and after treatment are shown in Figure 4.

**FIGURE 5:**
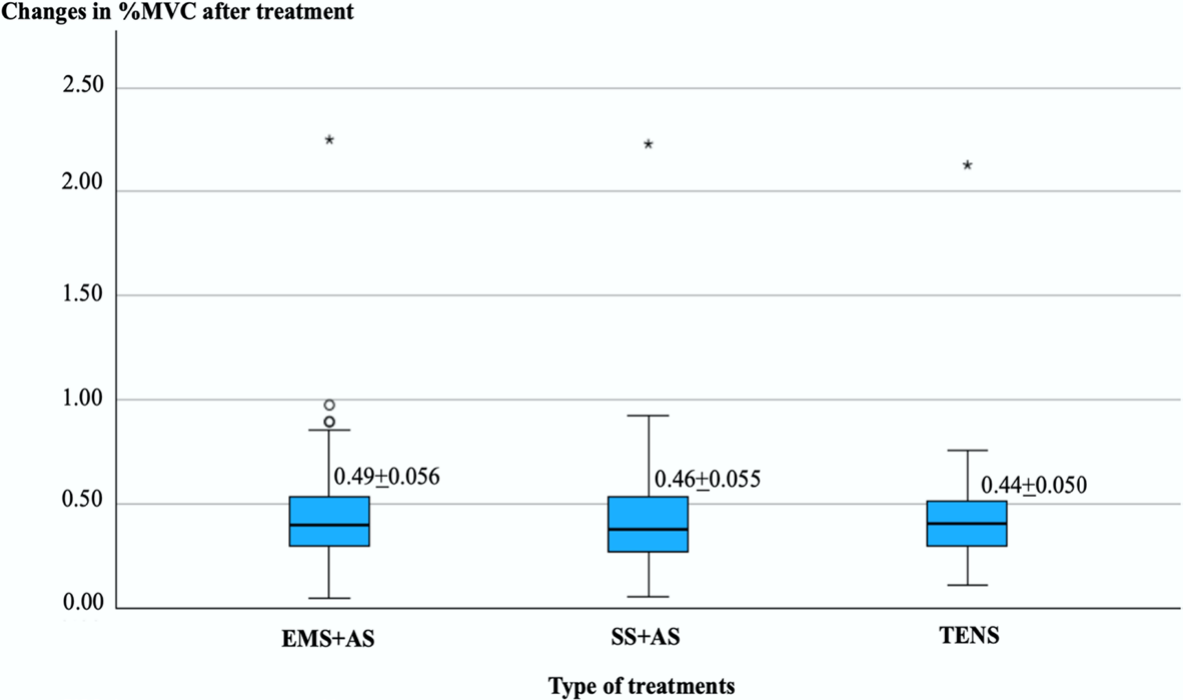
Illustration of changes in %MVC after EMS + AS, SS + AS, and TENS treatments (*Extreme outlier and °mild outlier). [Color figure can be viewed at wileyonlinelibrary.com]

A mean % MVC at post‐treatment normalized with pre‐treatment values of EMS + AS showed a higher value than SS + AS and TENS, as shown in Figure 5. However, there was no significant difference among the three treatments (*F*_(2,120)_ = 0.177, *p* = 0.838).

## 4 Discussion

Forty‐one participants with predominantly latent MTPs (73.2%) completed the study. Despite not causing constant pain, latent MTPs can contribute to TM dysfunction [5]. Participants reported moderate pain (6.22 ± 1.33) even though the self‐reported average hours of daily technology (6.71 ± 3.14 h) use were slightly less than the 7‐h average for office workers [15]. EMS + AS significantly reduced PI and increased PPT, indicating improved pain perception and potential mechanical changes within MTPs. Compared to SS + AS and TENS, EMS + AS demonstrated significantly greater PPT increases, suggesting enhanced pain relief beyond stretching alone. This study demonstrates the potential of EMS + AS in managing MPS.

Unlike the methodologies of EMS in previous studies [22, 23], our study aimed to use EMS to generate a localized contraction of the TM. This method of EMS + AS mimics the mechanism of PS with a therapist. In traditional therapist‐assisted PS, specific techniques are used to stabilize the body and maximize the stretch on the targeted muscle. For instance, with MTPs in the upper TM, a therapist stabilizes the scapula (at the acromion) with one hand while gently pushing the patient's neck in the opposite direction, creating a stretch. Similarly, for MTPs in the middle TM, the therapist stabilizes the spine's midline and pulls the patient's arm towards the body. Subject‐performed AS can also stretch the TM. However, the effectiveness of AS is often limited. This is because, without external stabilization, the scapula tends to move with the stretch, reducing the effective stretch on the MTP‐containing muscle region compared to PS. To overcome this limitation, our study strategically placed EMS electrodes with a small IED transversely across the muscle fiber. This allowed for localized muscle stimulation through proper force generation [32], without considering peripheral nerve location or motor point targeting [33]. In this setup, EMS effectively mimics the therapist's stabilizing hand, generating resistance around the scapula. This resistance enhances muscle stretches during the subject‐performed AS, thereby improving the stretch's effectiveness on the targeted MTPs.

The EMS protocol, derived from prior studies [34], has been validated to have no adverse effects on humans [35]. The average magnitude of EMS elicited strong tetanic muscle contractions, which, before and during TM stretching, provided the mechanism for the therapeutic effect. The pain diminished when EMS was stopped, likely due to the sympathetic effects of electrical stimulation, including endorphin release [13, 14, 21]. This electrically induced muscle tension, particularly near the acromion or spine, generated by EMS allows Golgi tendon organs (GTOs)—proprioceptors located in the musculotendinous junction—to rapidly detect changes in tension. This muscle tension then triggers the lengthening reaction, mediated by spinal cord connections, which inhibits muscle contraction and promotes relaxation [24, 25]. This contract‐relax natural human spindle reflex, which helps prevent muscle injury from overstretching and excessive tension, is key to the therapeutic mechanism. Indeed, a previous study has demonstrated that PS exercise, consisting of three 30‐s repetitions, effectively relieves neck pain [36]. Our EMS + AS treatment, however, aims to achieve similar outcomes in an even shorter duration. The EMS + AS protocol was designed as an effective stretching technique lasting only 1 min (excluding the time required to locate and affix the electrodes). Individuals often rely on self‐care, such as AS, to alleviate TM pain. Our technique offers office workers a potentially more time‐saving and efficient alternative compared to AS alone. TM stretching effectively reduces muscle fiber tension and enhances blood circulation around the MTP [24, 25]. This mechanism aligns with the shift model, based on energy crisis theory, which suggests that reduced muscle loading decreases muscle contraction time and increases relaxation time. This shift in the contraction‐relaxation ratio minimizes MTP formation by reducing waste product accumulation and inflammatory mediator release, ultimately decreasing pain [37].

EMS + AS utilizes a different mechanism compared to TENS, which primarily involves central sensitization through nociceptors, as explained by the gate control theory. This approach typically requires a longer time for optimal pain relief [13, 14, 16, 18, 19]. TENS mechanisms, when applied for a limited duration, did not involve changes in muscle length at the MTP. Although TENS treatment can show immediate PI reduction, this approach typically requires more time to promote tissue healing at MTPs and improve PPT and muscle electrical activity during TM movement [19]. For the primary outcomes, EMS + AS significantly improved PI and PPT in just 1 min, demonstrating its acute effectiveness for MPS. Moreover, the effects of EMS + AS showed better outcomes than SS + AS and TENS. This suggests that EMS + AS can be a valuable adjunct to routine daily self‐stretching for MPS management.

For the secondary outcome, EMG signal interpretation requires careful consideration due to the complex pathophysiology of MTPs. EMG amplitude reflects muscle force, while MTPs limit TM function [38], impacting performance [30]. The percentage changes in %MVC after EMS + AS treatment were minimal, likely due to the brief treatment duration, as it was not a full therapy session. These changes may not yet be clinically meaningful, but a complete therapy session may demonstrate more pronounced %MVC changes. Although a slight increase in %MVC was observed, the effect of EMS + AS likely involved pain reduction and improved sEMG activity during TM movement. This is because the MTP was less prone to generating obstructions, aligning with research demonstrating that even short durations of stretching (e.g., 60 s) can improve muscle fiber organization after trigger point‐induced distortions [39].

However, the MTP did not disappear when observed via palpation of the snapping nodule after treatment. This implied that a 1‐min treatment had only delayed the progression of MTPs, as sarcomeres tend to return to their original state after immediate muscle relaxation [5, 6]. Therefore, EMS + AS may offer immediate relief for upper and middle TM trigger points; however, long‐lasting benefits require further investigation. Typically, a full therapy session involving AS or PS requires about 3–5 rounds [26, 36]. For EMS + AS, a full therapy session should similarly include 3–5 rounds. To achieve sustained benefits and prevent future MTP formation, this should be combined with prescribed stretching multiple times a day (e.g., twice daily) [25] or several days a week (e.g., 3–4 days per week) [24]. Each round of EMS + AS lasts 60 s (i.e., stretching for 10 s, relaxing the muscle for 10 s, repeating three times until reaching the new endpoint).

One limitation of this study was that the acute effects of EMS + AS, TENS, and SS + AS were assessed for approximately 1 min of treatment before a 3–5‐min full session of EMS + AS treatment. However, the full therapeutic benefit from TENS is typically experienced over a 20‐min session, and so comparisons may be limited. Further, several studies have recommended *combination* treatments to manage MTPs, and so it is likely that EMS + AS combined with myofascial release therapy may further improve outcomes [5, 6, 11, 12]. The data should be segmented by TM section and MTP type for more precise results. Although careful repositioning of the sEMG sensor at the same location was intended to ensure consistency, it may have affected amplitude interpretation. However, the data were normalized to %MVC to allow comparison across treatments [31]. Additionally, the effectiveness of the EMS + AS technique in improving flexibility or releasing MTP knots in tight muscles remains uncertain. While PS has been shown to improve flexibility, it does not typically resolve MTP knots [26, 36], unlike single dry needling [38] that is capable of inducing reversibility in MTPs [12] and significantly increasing sEMG activity when TM is active. Therefore, a subsequent investigation will assess ROM and utilize ultrasound imaging to provide supplementary information on morphological changes within the MTP. In addition, the effects of EMS + AS and PS will be compared in a further study.

In conclusion, the findings of this study suggest its potential as a novel approach. EMS + AS significantly improved PI and PPT between pre‐ and post‐treatment, and this treatment improved the mean %MVC of amplitude analysis during TM function. When comparing SS + AS and TENS within a one‐minute treatment protocol, EMS + AS briefly showed significant improvement in PPT. However, there were no significant improvements in PI or the mean %MVC of amplitude analysis during TM function across these three treatments. Future research should compare EMS + AS to PS alone, including assessments of ROM and MTP size, to elucidate treatment mechanisms.

## Author Contributions

S.C. designed the experimental protocol, conducted the experiments, analyzed the data, and drafted the manuscript. B.M. helped analyze the data and edit the manuscript. P.M. helped design the experimental protocol. D.Z. conceived of the study, co‐designed the experimental protocol, and co‐drafted the manuscript. All authors read and approved the final manuscript.

## Conflicts of Interest

The authors declare no conflicts of interest.
